# Detection and analysis of stable and flexible genes towards a genome signature framework in cancer

**DOI:** 10.6026/97320630015772

**Published:** 2019-11-10

**Authors:** Emir Sehovic, Adem Hadrovic, Senol Dogan

**Affiliations:** 1 International Burch University Sarajevo, Francuske Revolucije BB, 71210 Sarajevo; 2 Sarajevo School of Science and Technology, Hrasnicka Cesta 3a, 71210 Sarajevo; 3 The University of Leipzig, Faculty of Physics and Earth Science, Peter Debye Institute for Soft Matter Physics, LinnestraBe 5, 04103 Leipzig, Germany

**Keywords:** Cancer, pattern analysis, cancer types, statistics, model, gene expression, stable, flexible

## Abstract

Comparison and detection of stable cancer genes across cancer types is of interest. The gene expression data of 6 different cancer types (colon, breast, lung, ovarian, brain and renal) and a control group from The Cancer Genome Atlas (TCGA) database were used in this study. The comparison of gene expression data together with the calculation standard deviations of such data was completed using a statistical model for the detection of stable genes. Genes having similar expression (referred as flexible genes) pattern to the control group in four out of six cancer types are PATE, NEUROD4 and TRAFD1. Moreover, 13 genes showed low difference compared to the control group with low standard deviation across cancer types (referred as stable genes). Among them, genes GDF2, KCNT1 and RNF151 showed consistent low expression while ODF4, OR5I1, MYOG and OR2B11 showed consistent high expression. Thus, the detection and analysis of stable and flexible cancer genes help towards the design and development of a framework (outline) for specific genome signature (biomarker) in cancer.

## Background

**Figure 1:**
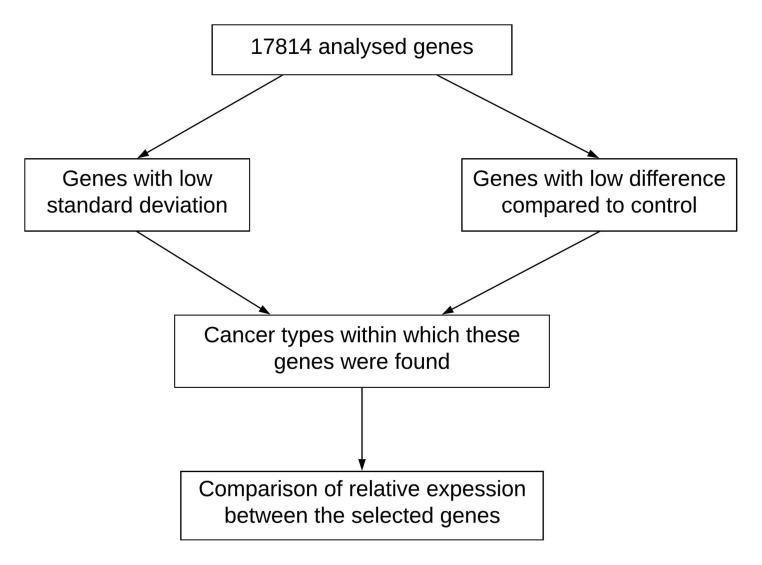
Flowchart for the detection and analysis of stable and flexible genes towards a genome signature framework in cancer.

Cancer is a disease that is manifested through the uncontrollable growth of cells and their proliferation to tissues in other parts of the body [1]. Since the diversity of gene expression is very large across cancer types, it is difficult to consistently find the same dys-regulated genes. Gene expression has been used for profiling cancer types and subtypes [2-4]. Moreover, gene expression is generally compared between individuals with cancer and a control group of healthy individuals. The use of computer aided statistics models for the analysis of biological data generated using Next Generation Sequencing (NGS) techniques along with gene expression, methylation, microRNA expression and mutational profiles have become common [5-7]. The use of whole genome sequencing data in personalized therapies is gaining momentum in recent years [8-10].It is known that epigenetic factors such as methylation, microRNA expressions and mutational ffect the gene expression profile in many cancer types [11-13]. Computer aided statistical analysis of cancer genomes for establishing the potential correlation among gene expression in cancer is getting frequent in current research and development [14,15]. Therefore, it is of interest to report stable and flexible gene expression patterns in cancer cells [16]. Hence, we describe a statistics model to identify stable and flexible genes among six different cancer types (colon, breast, lung, ovarian, brain and renal) as shown in Figure 1.

## Methodology

### Dataset:

The dataset used in this analysis consists of microarray gene expression values obtained from the TCGA gene expression database [17]. The dataset analysed involved six cancer types and one control group. The analysed cancer types are colon, breast, brain, lung and ovarian and renal cancer. All six cancer types, as well as the control group, had the same number of 17814 genes for the analysis. The dataset included colon, breast, brain, lung, ovarian, and renal cancer types with 174, 621, 694, 32, 255 and 72, respectively. It should be noted that data from 1896 individuals were represented in the study with 48 of them in the control group.

### Methodology and Statistical Analysis

Descriptive statistics, which involved the mean, standard deviation and fold, was calculated in the IBM SPSS Statistics 23 program. The mean, as well as the standard deviation, was calculated for each gene within the six cancer types and the control group. Afterward, the average of the standard deviation of each gene and the standard deviation of the averages of gene expressions were calculated. Data used in the analysis is not normally distributed. Hence, Mann Whitney's U test was used to compare the medians of the expression data between the analysed cancer types and control groups.

Fold values were calculated for all the genes within all six cancer types. The criteria for selecting the genes of interest were 0.1% of the genes from each cancer type with the lowest standard deviation as well as the lowest difference when compared to the control group. The same criteria were used for genes of interest with a high difference in expression. Afterward, the common genes among the six cancer types were selected. The genes were sorted based on the number of cancer types they repeated in (ranging from 2 to 6). Three categories of repeating genes that met the required criteria were made. One category included the common genes within the cancer types with low differences in gene expression compared to control group. The second one included the common genes with low standard deviation within the cancer types. Finally, the third group included the common genes, within the analysed cancer types, with a large difference in expression compared to the control group and a small standard deviation of gene expression. For the sake of further comparison and analysis a list of genes with a low standard deviation of gene expression within the control group was also created.

Sorting of gene expression values as well as finding the common genes within the above-mentioned criteria was performed in a custom-made Python script. When the Python script is run, it takes an input .csv file that contains a table of genes that were found in cases of colon, ovarian, breast, lung, brain, and renal cancer, as recorded in the data mentioned above. The input .csv files used contained data for the recorded common genes with low difference in gene expression within the cancer type, data for the recorded common genes with low standard deviation within the cancer types, and data for the recorded common genes with both low differences compared to the control group and low standard deviation, respectfully. The script continues by finding the presence of each gene across all the cancer types and sorts the data. To achieve this, a list of unique gene values is assembled. Then, for each unique expression, the table data is scanned for occurrences in each column. If a gene expression is present in a column, the column number is appended to the results for that gene, where 0 is the first column instead of 1 and the column order matches the order of the aforementioned cancer types. A sample result for a gene would look something like; Gene X: 2 3 4 0. This result would be interpreted as; Gene X was present in Breast, Lung, Renal, and Colon cancer. Once finished, a formatted text file is generated with the results. Details, as well as the code of this custom-made script, are given in the supplementary material(See PDF File).

Average gene expression data of selected genes was analysed and compared through a heat map figure using the HCE 3.5 program [18]. The same program was used to visualize hierarchical clustering based on Euclidean distance of the gene expression values. Moreover, to determine the function of selected genes as well as their interaction with each other and other relevant genes the GeneMANIA database [19] was utilized. The key interaction categories analysed were co-expression, shared protein domains, co-localisation, pathways and physical interactions.

## Results

**Figure 2:**
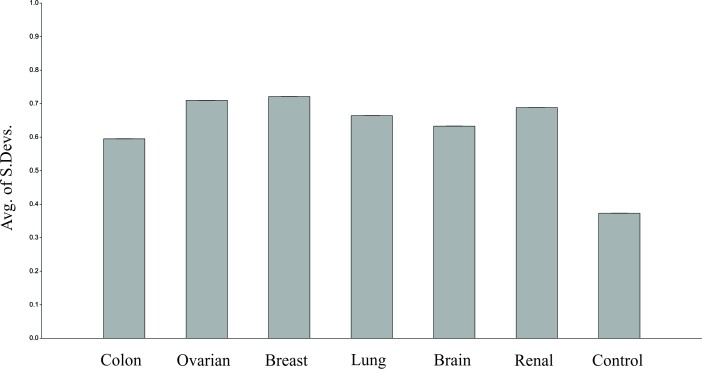
The mean standard deviations for all cancer types to depict stable genes on a particular group.

All six analysed cancer types have a larger overall expression mean when compared to the control group. The average of the standard deviations of analysed genes within all cancer types differs significantly when compared to the control group. The largest average standard deviation of gene expressions is found within breast cancer type (0.721) and lowest within colon cancer type (0.595). Hence, as can be seen in Figure 2 the average standard deviation of gene expressions in the control group is much lower than the colon cancer type. Mann-Whitney U test was performed on analysed cancer types compared to the control group. All 6 cancer types have differed significantly. The p-values obtained are all lower than 0.001.

Genes that have a very similar expression pattern to the control group and appear as such in 4 out of six cancer types are PATE, NEUROD4 and TRAFD1. PATE is found to have a very low difference in gene expression compared to the control group in colon, breast, brain and renal cancer types. Furthermore, NEUROD4 is found to have a very low difference in expression compared to the control group in colon, ovarian, breast and lung cancer types. Finally, TRAFD1 is found to have a very low difference in expression compared to the control group in colon, ovarian, brain and renal cancer types.

A total of 211 genes with very low relative standard deviation in gene expression which repeated in all 6 cancer types were found (data not shown). Genes which have the lowest relative standard deviation when all six cancer groups are analysed individually and also have a low standard deviation in all six cancer groups are NXNL1, PATE, C21orf89, OR10G7, CSHL1, GRM2, OR10A5, OR8H1, OR1A1, NHLH2, EIF2B1, OR7D4, CRHR1, INHBC, PGLYRP1, OR6N1, OR13F1, ATP1B4, OR10A4, TNP2, C7orf42, TP73, TAS2R60 and STX10. All of these genes, except for PATE, have a low standard deviation of gene expression within the control group.

Genes that have a low difference in gene expression in at least 4 cancer types compared to the control group and a very low standard deviation in all 6 analysed cancer types are OR5I1, GRM2, GDF2, MYOG, OR2AG1, OR2B11, CRHR1, NTSR1, ZNF645, CBLN3, ODF4, KCNT1, RNF151. None of the genes are found to have a very low difference in expression in all six cancer types. The list of those genes, as well as the cancer types in which they have met the set criteria, can be found in Table 1.

Genes with a large difference in gene expression when compared to the control group as well as a low standard deviation are selected for further analysis. TMEM125, C1orf172 and KLHL9 are the 3 genes that are found in more than one cancer type.

**Figure 3:**
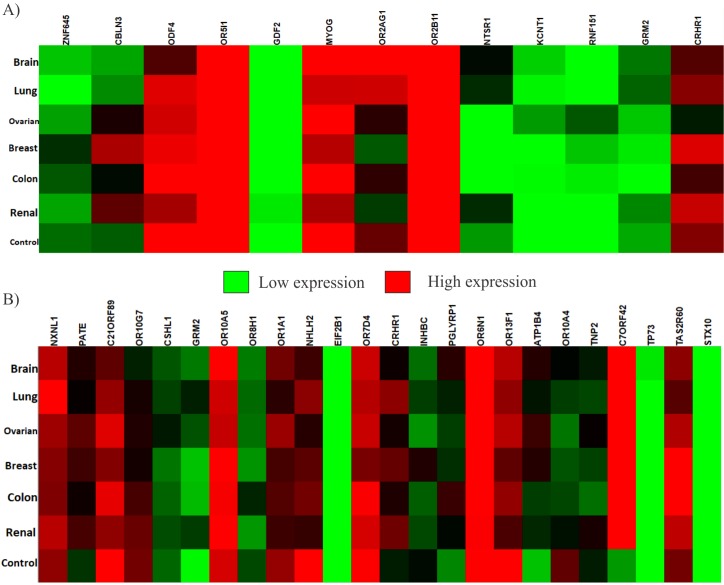
Heat map for relative gene expression of the genes within cancer types and the control group. (A) Genes that are listed in Table 1. (B) Genes, which have a low standard deviation in all cancer types.

**Figure 4:**
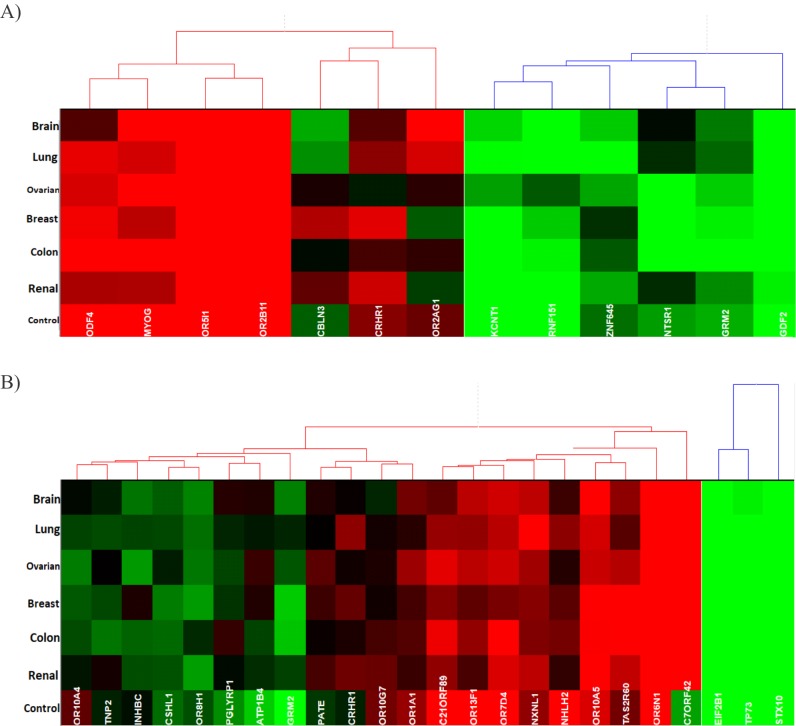
Hierarchical clustering based on the Euclidean distance of analysed gene expressions within the cancer types and the control group. (A) Genes that were listed in Table 1. (B) Genes which have a low standard deviation in all cancer types.

Relative to each other, among the genes listed in Table 1, the genes which consistently, among all six cancer types, have a lower expression are GDF2, KCNT1 and RNF151. Genes KCNT1 and RNF151 are also very close in the hierarchical clustering based on the Euclidean distance as can be seen in Figure 4. On the other hand, the genes that consistently have a higher expression are ODF4, OR5I1, MYOG and OR2B11. All four of these genes cluster together in the Hierarchical clustering. The relative gene expression values have been presented through a heat map in Figure 3.

Within the set of genes that have a low standard deviation in all six-cancer types the genes that consistently have a lower expression relative to each other are EIF2B1, TP73 and STX10. The genes with a high relative expression within the mentioned set are OR10A5, OR7D4 and OR6N1. In this case, the genes that consistently have a lower expression cluster in a more uniform fashion than the genes, which consistently have a higher expression. Details can be seen in Figure 3. The gene C7ORF42 has a relatively higher expression within all cancer types excluding the control group when compared to other genes in the respective set. This gene seems to have relatively lower expression than that of the other genes in the set within the control group.

According to GeneMANIA [19], there is an overall 75.09% co-expression between the analysed genes from Table 1 (Figure not shown). Furthermore, there are overall 24.11% shared protein domains and 0.80% gene interactions. Within these 13 genes, it is found that NTSR1 tends to co-express with CBLN3 and OR51I while the gene CBLN3 co-expresses with MYOG and NTSR1 genes. The gene GDF2 co-expresses with MYOG and KCNT1. Genetic interactions are found between O2AG1 and GRM2 as well as ZNF645 and CRHR1 genes. Shared protein domains are found between genes ZMF645 and RNF151.

The interaction results of PATE, NEUROD4 and TRAFD1, which have a very similar expression pattern to the control group, have shown a considerable number of genes with which they interact (data not shown). The main interactions analysed are physical co-expression, pathways, shared protein domains and co-localisation. PATE1 co-expresses with NEUROD2. NEUROD4 has shared protein domains with NEUROD6, NEUROD2 and NEUROD1. Furthermore, it has physical interactions with LRRN2 and GABRB1. It also co-expresses and has shared pathways with the gene GCM2. The gene TRAFD1 has shared protein domains with genes TRAF1, TRAF2 AND TRAF3. It co-expresses with genes TICAM1 and TRIM21. Moreover, it has physical interactions with genes TRAF6, UBC, PAN2, NGLY1, CDK20, FAM46A, GET4 and ILK.

## Discussion

All cancer types have a larger average expression than the control group, and the medians of all cancer types are significantly different when compared to the control group. The control group has the lowest average standard deviation of gene expression. The highest average standard deviation of gene expression among cancer types, was observed within the breast cancer type while the smallest within the colon cancer type. The control group has the lowest average gene expression where lung cancer has the largest and the ovarian cancer has the smallest average gene expression. On average, the analysed six cancer types and the control group have a similar overall standard deviation calculated on all expression values. However, major differences in average standard deviation values and individual expression patters of genes between the control group and the cancer types, as well as between the cancer types themselves were observed. Studies have been successful in finding and identifying potential cancer driving genes [20]. Similarly, we have found genes that have stable expression patterns, as such and they could be linked to cancer.

The genes selected for further investigation are PATE1, NEUROD4 and TRAFD1. Due to their low difference in gene expression value when compared to the control group, they might be involved in functions, not altered by cancer and which could be essential in sustaining the survival of cancer cells. Moreover, PATE1 is also found to have a very low standard deviation of gene expression within all cancer types. These three genes have a broad spectrum of functions and have few similarities with each other. PATE1 is involved in sperm-egg penetration and sperm motility [21]. Moreover, gene NEUROD4 is thought to act as a transcriptional activator as well as a mediator in neuronal differentiation [21]. TRAFD1 is involved in negative feedback regulation that controls innate immune responses [21].

Genes that have a very low difference in gene expression compared to the control group and low standard deviation could also be genes which are conserved within cancer types and have a function which does not tolerate unstable expression, possibly regulated by nuclear lamins [22] which are believed to have a role in protecting the cancer genome [23], and is essential for the proliferation of cancer. Similar genes might be useful in designing better models for predictive, diagnostic or prognostic tools based on expression profiling [24]. Moreover, changes in gene expression in cancer cells are sometimes correlated to epigenetic regulations [13]. Hence, the epigenetic structure of DNA regions in which stable genes are found could be a potential research focus to better understand stably expressed genes without significant alterations.

The bolded genes in Table 1 (GRM2, CRHR1, CBLN3 and ODF4), which also have a low standard deviation within the control group, could be very important for cancer proliferation. GRM2 codes for L-glutamate, which is one of the major, neurotransmitters in the central nervous system and activates both iono tropic and meta botropic glutamate receptors [21]. The gene CRHR1 encodes a G-protein coupled receptor that binds neuro peptides regulating the hypothalamic-pituitary-adrenal pathway [21]. CBLN3 gene is considered to be involved in synaptic functions [25]. Finally, ODF4 encodes a protein that is believed to have an important role in the sperm tail [21].

Out of the 13 genes in Table 1, five genes repeated in colon, ovarian and lung cancer types simultaneously while 3 genes repeat simultaneously in colon, breast and lung cancer types for further evaluation. The genes that had a high expression when compared to the control group, low standard deviation and repeat in multiple cancer types are TMEM125, C1orf172 and KLHL9. Clorf172 is mainly responsible for the regulation of epidermis formation during early development [25] while the gene KLHL9 is responsible for coordinating mitotic progression and cytokinesis completion [26,27].

Some of the functions of these genes are G-protein coupled receptor activity, potassium ion transport, mono valent inorganic cation transport, calcium-activated potassium channel activity and other functions related to channel and transport activity which were found in the gene interaction network available at GeneMANIA [19]. Most of the functions of genes in Table 1, analysed within the gene interaction network, seem to be connected to various channel activities. The gene interactions analysed between genes that have a very similar expression pattern to the control group are involved in regulation of NIK/NF-kappaB signalling, T cell cytokine production, positive regulation of production of molecular mediator of immune response, regulation of transcription regulatory region DNA binding, activation of NF-kappaB-inducing kinase activity and other functions related to NF-kappaB signalling [19].

## Conclusion

It is of interest to report genes whose expression does not considerably change in cancer cells when compared to the control group having stable expression patterns with low standard deviation. We further relate these genes with known functions in cancer or normal cells. These genes are often liked to membrane channel functions involving NF-kappa B signalling. Thus, a framework for a pattern of gene expressions that are relatively stable across different types of cancer is described in this report requiring further validation using an updated dataset with more classification for improved clarity in future studies.

## Figures and Tables

**Table 1:** Genes that have very low difference in expression in at least 4 cancer types compared to the control group with low standard deviation in all cancer types. Bolded genes also have a low standard deviation of gene expression within the control group.

Gene	Cancer type (low gene expression difference)
ZNF645	Colon, Ovarian, Breast, Renal
CBLN3	Colon, Ovarian, Lung, Brain
ODF4	Colon, Ovarian, Breast, Lung
OR5I1	Colon, Breast, Lung, Brain
GDF2	Ovarian, Breast, Lung, Brain
MYOG	Colon, Ovarian, Lung, Brain
OR2AG1	Colon, Ovarian, Lung, Renal
OR2B11	Colon, Ovarian, Lung, Brain
NTSR1	Colon, Ovarian, Lung, Brain
KCNT1	Colon, Breast, Lung, Brain
RNF151	Breast, Lung, Brain, Renal
GRM2	Colon, Ovarian, Breast, Brain
CRHR1	Colon, Breast, Lung, Brain

**Table 2:** Genes that have a large mean difference when compared to the control group with low standard deviation is given. The common genes among the cancer types are shown in bold.

Colon	Ovarian	Breast	Lung	Brain	Renal
TMEM125	TMEM125	CD93	C1orf172	ZNF502	GPATCH1
C1orf172	KLHL9		CMTM8	APCDD1	KCNH4
KLHL9	WFDC2			FXYD6	AHCYL1
KRTCAP3	ZNF239				AQP8
SPINT2					CD209
CLDN4					FNTB
					C3orf39
					MXD1
					SUHW4
